# Boosting Dendritic Cell Function in Cancer

**DOI:** 10.1002/cam4.71062

**Published:** 2025-08-28

**Authors:** Evangelos Giampazolias

**Affiliations:** ^1^ Cancer Immunosurveillance Group, Cancer Research UK Manchester Institute The University of Manchester Manchester UK

**Keywords:** activation, antigen presentation, cancer immunity, dendritic cells, immunotherapy, T cell priming

## Abstract

Instruction of T cell immunity is a key function of sentinel leukocytes called dendritic cells (DC). Several studies in mice and humans have demonstrated a key role for DCs in promoting T cell responses to cancer and augmenting the efficacy of T cell‐based immunotherapies. Like other innate immune cells, DCs express a wide repertoire of receptors endowing them with the ability to detect microbial presence and tissue damage. These functions contribute to cancer immunity and have been previously linked to the induction of anti‐tumour CD8^+^ T cells and enhanced responses to immune checkpoint blockade (ICB) therapy. Here, I review some of the principles of DC biology, highlighting their functional characteristics that dictate T cell responses to cancer and how these can be harnessed in the design of novel immunotherapies.

## 1 Introduction

Around 30 years ago, seminal studies in mice identified components of the immune system capable of preventing the development of spontaneous and carcinogen‐induced tumours [1, 2, 3]. Since these early studies, it is now well established that the anti‐tumour function of the immune system is largely dependent on cytotoxic CD8^+^ T lymphocytes (CTL) that directly kill cancer cells through recognition of tumour antigens that are presented alongside major histocompatibility complex (MHC) class I molecules. The cytolytic activity of CTLs is negatively regulated by inhibitory receptors that are present on their surface, acting as immune checkpoints [4, 5]. Immune checkpoint blockade (ICB) therapies are a type of T cell‐based immunotherapy that targets such receptors, for instance, using blocking antibodies to anti‐CTLA‐4 and anti‐PD‐1, and are currently used as a frontline treatment in patients with several advanced cancers. Despite remarkable clinical outcomes, most cancers continue to exhibit significant heterogeneity in response rates, and durable clinical benefits are limited to a small subset of patients [6]. Resistance to ICB is often attributed to blunted CTL activation and intratumoural infiltration [6]. Therefore, deciphering the mechanisms that govern effective CD8^+^ T cell immunity is of paramount importance to overcome immunotherapy resistance.

Dendritic cells (DCs), known as the sentinel cells of the innate immune system, are now well recognised for their non‐redundant role in orchestrating T cell immunity to host threats, including cancer. In this review, I will first define DCs based on their origin and then describe their functional properties that shape cancer immunity. I will further discuss the cell‐intrinsic and extrinsic determinants of DC‐mediated cancer immunity identified using mouse models and consider strategies to overcome immunotherapy resistance by harnessing the therapeutic potential of DCs in cancer.

## 2 Defining Dendritic Cells

**FIGURE 1:**
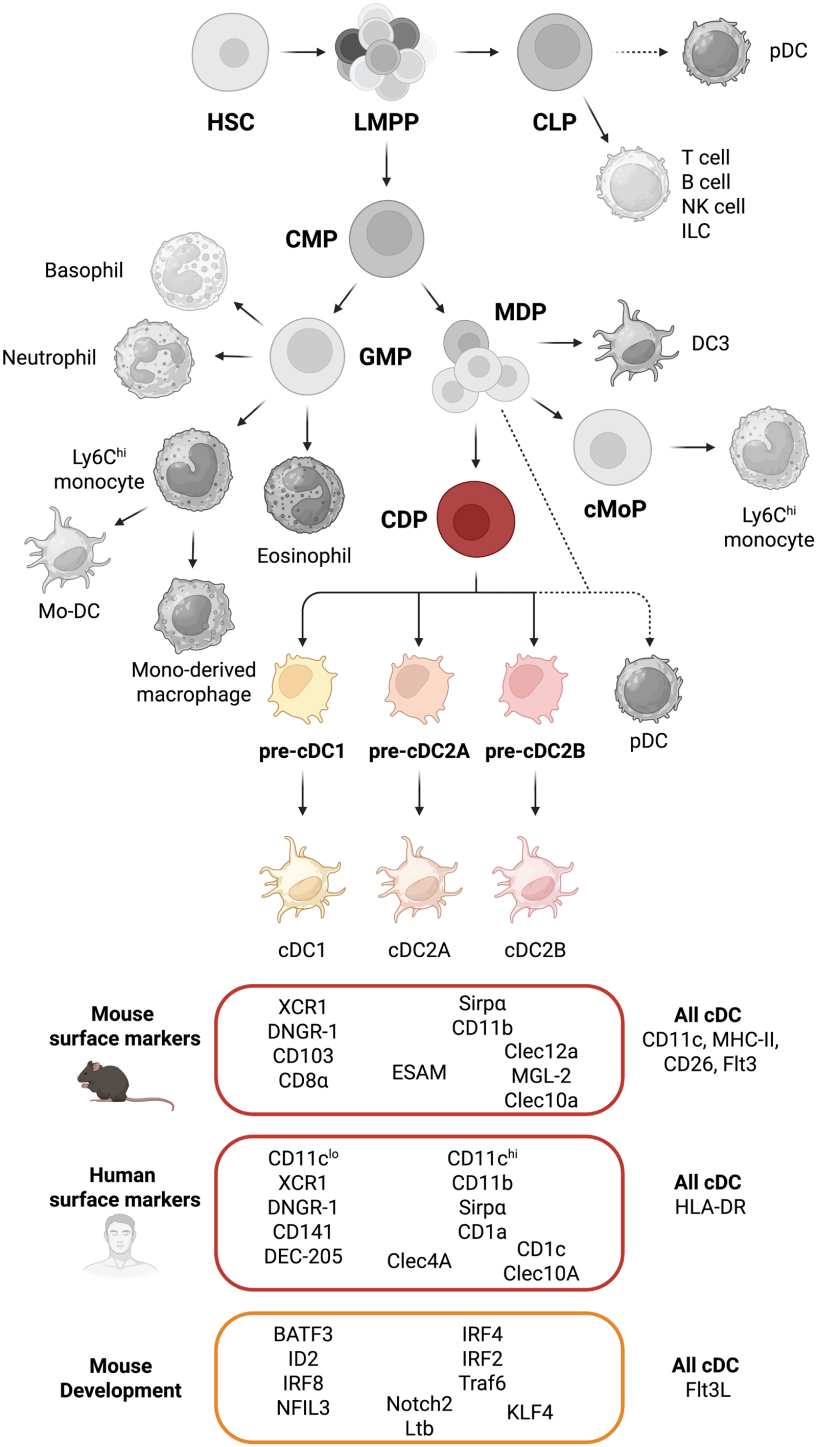
Haematopoietic origin of conventional dendritic cells. cDCs develop in the bone marrow from haematopoietic precursors. The common dendritic cell precursor (CDP) is the first progenitor with exclusive potential to generate the cDC lineage. pDC may also derive from CDP, but their origin is a matter of debate (dotted lines) as they might also arise directly from the monocyte‐dendritic cell precursor (MDP) or have lymphoid origin, arising from the common lymphoid progenitor (CLP). CDP gives rise to committed pre‐cDC subsets that leave the bone marrow and seed tissues where they locally generate cDC1, cDC2A and cDC2B. Surface markers and key factors that regulate all cDC or subset‐specific development are listed. Cells belonging to the cDC lineage are depicted in colour, whereas all other lineages are depicted in grey. cMoP, common monocyte progenitor; CMP, common myeloid progenitor; GMP, granulocyte‐monocyte progenitor; HSC, haematopoietic stem cell; LMPP, lymphomyeloid‐primed progenitors.

DCs, named after the Greek *dendron*, meaning tree, were initially identified by Ralph Steinman and Zanvil Cohn more than 50 years ago, as rare phagocytic cells in mouse spleen with distinct stellate shape that, unlike macrophages, were capable of stimulating T cell responses following antigen uptake and presentation [7]. Flow cytometry and gene expression analysis coupled with fate‐mapping mouse models and single‐cell technologies paved the way for the identification of distinct DC subpopulations that have been categorised on the basis of cellular origin [8, 9]. Conventional DCs (cDCs) are derived from bone marrow progenitors with cDC‐restricted potential called pre‐cDC [10] (Figure 1). Two major cDC subtypes have been described, type 1 (cDC1) and type 2 (cDC2) cDC, whose development is dependent on the growth factor FMS‐like tyrosine kinase 3 ligand [8] (Flt3L). The development of cDC1 and cDC2 is dependent on differential transcription programmes, some of which become active at the progenitor level and impose commitment to a specific cDC fate [11, 12, 13]. cDC1s can be identified by the expression of specific surface markers [8], the chemokine receptor XCR1 and the C‐type lectin receptor CLEC9A (aka. DNGR‐1) and their differentiation depends on *Irf8, Batf3, Nfil3* and *Id2* [10, 11] (Figure 1). cDC2s specifically express Sirpα and CD11b, and the development of some cDC2s depends on *Irf4* and *Irf2* transcription factors and the adaptor molecule Traf6 (Figure 1). In contrast to cDC1s, cDC2s are a much more heterogeneous subset comprising cells with different developmental programmes [9]. Two subsets of cDC2 with discrete bone marrow progenitors have been identified to be conserved in mice and humans [14], which depend on Notch2 receptor and transcription factor Klf4, corresponding to Tbet^+^ cDC2As and Tbet^−^ cDC2Bs [11, 14], respectively (Figure 1).

In addition to origin, cDC subsets have also been classified on the basis of function. Resting cDCs in epithelial surfaces and lymphoid organs continuously sample cell‐associated and soluble material via phagocytosis and micropinocytosis [9]. At steady state, cDCs promote immune tolerance to self, dietary and commensal antigens by suppressing reactive T cells and averting immune responses that can be harmful to the host, including autoimmunity, allergy and dysbiosis, respectively. During infection or inflammation, triggering of innate immune receptors by microbial or damage‐associated molecular cues can lead to cDC activation. Activated cDCs acquire the capacity to communicate with neighbouring cells and contribute to eliminating host threats by instructing T cell responses within local lymphoid tissues. In addition to T cell priming, activated cDCs can interact with other immune and non‐immune cells, modulating their activity at steady state or in response to noxious substances [15, 16, 17]. cDC1s produce interleukin (IL)‐12 and are often indispensable for the cross‐presentation of exogenous antigen to CD8^+^ T cells, leading to host elimination of intracellular pathogens and tumours [18, 19]. In turn, cDC2s might be more relevant to the instruction of host responses to extracellular pathogens such as bacteria, parasites and fungi by priming CD4^+^ T cells [9]. This functional categorisation could be explained by their origin and differential localisation of cDC subsets in lymphoid tissues, thereby determining antigen access and interactions with T cell subsets [20], as well as by subset‐specific mechanisms of antigen acquisition and processing [9]. However, these roles are not mutually exclusive, as cDC1s can also prime CD4^+^ T cells [21, 22] and upon exposure to type I interferon (IFN‐I), cDC2s can induce IRF8 expression, permitting IL‐12 secretion during viral infection [23]. In some instances, cDC2s have also been reported to display non‐redundant functions in priming CTL responses to transplantable tumours [24]. Nevertheless, human cDC2s, unlike their mouse counterparts, produce IL‐12 and are capable of engulfing and cross‐presenting dead cell‐associated antigen, which all contribute to CTL priming, arguing for differences in cDC subset function across species [25, 26, 27]. Collectively, these findings suggest that there is functional plasticity in cDCs that can override ontogenetic programming in several instances.

Several other immune populations, including DC3s that descend from Ly6C^+^ monocyte‐dendritic cell progenitors (MDP), monocyte‐derived dendritic cells (Mo‐DCs) and lymphoid‐derived plasmacytoid cells (pDCs), have been previously classified as dendritic cells due to phenotypic similarities to cDCs [9]. These cells share phenotypic markers with cDCs; although they can participate in several aspects of pro‐ or anti‐tumorigenic inflammation, they exhibit limited naïve T cell priming capacity [28, 29]. Recent genetic profiling and use of lineage‐tracing models revealed a distinct monocytic origin of Mo‐DCs and DC3s compared to cDCs [23, 30, 31]. In contrast, the classification of pDCs as dendritic cells still remains a subject of debate [32, 33]. In this review, I use the term “cDCs” exclusively to describe cDC1s and cDC2s, excluding other phenotypically similar cells, as these cells are uniquely capable of naïve T cell priming across both infection and cancer.

## 3 Dendritic Cells in Cancer Immunity

In multiple human cancers, gene signatures that reflect cDC1 intratumoural abundance positively correlate with increased patient survival and responsiveness to ICB therapy [15, 34, 35]. Similarly, a high cDC2 gene signature on its own or in conjunction with a lower T regulatory (Treg) cell signature was found to be associated with improved outcomes for patients with luminal breast cancer and head and neck cancer, respectively [35, 36]. Although human studies have provided strong correlative evidence for the contribution of cDCs to anti‐cancer immunity, the establishment of the critical role of cDCs in cancer immunity came from loss‐of‐function studies in mice. Mice lacking the transcription factor Batf3 or an enhancer 32 kilobases (kb) downstream of the Irf8 transcriptional start site lack cDC1 development and are unable to reject immunogenic transplantable tumours [19, 37]. Similarly, diphtheria toxin‐mediated cDC1 depletion in *Xcr1* ^*DTR*^ mice bearing established tumours inhibits the response to T cell‐based immunotherapies [38]. Further studies in mice have highlighted functional redundancies between cDC1s and cDC2s in promoting anti‐cancer immune responses [24, 36, 39, 40, 41]. However, in most instances, cDC2s are not sufficient to compensate for the loss of cDC1s and fail to drive immune‐mediated tumour control, highlighting a context‐dependent advantage of cDC1s in manifesting immunity to cancer [19, 37, 38]. Whether cDC2s exert specialised, non‐redundant anti‐cancer functions is still unclear due to the lack of genetic mouse models that specifically lack cDC2s. Collectively, in both mice and humans, cDC1 and potentially cDC2 are critical players in driving anti‐cancer immune responses and ensuring the efficacy of T cell‐based immunotherapy.

**FIGURE 2:**
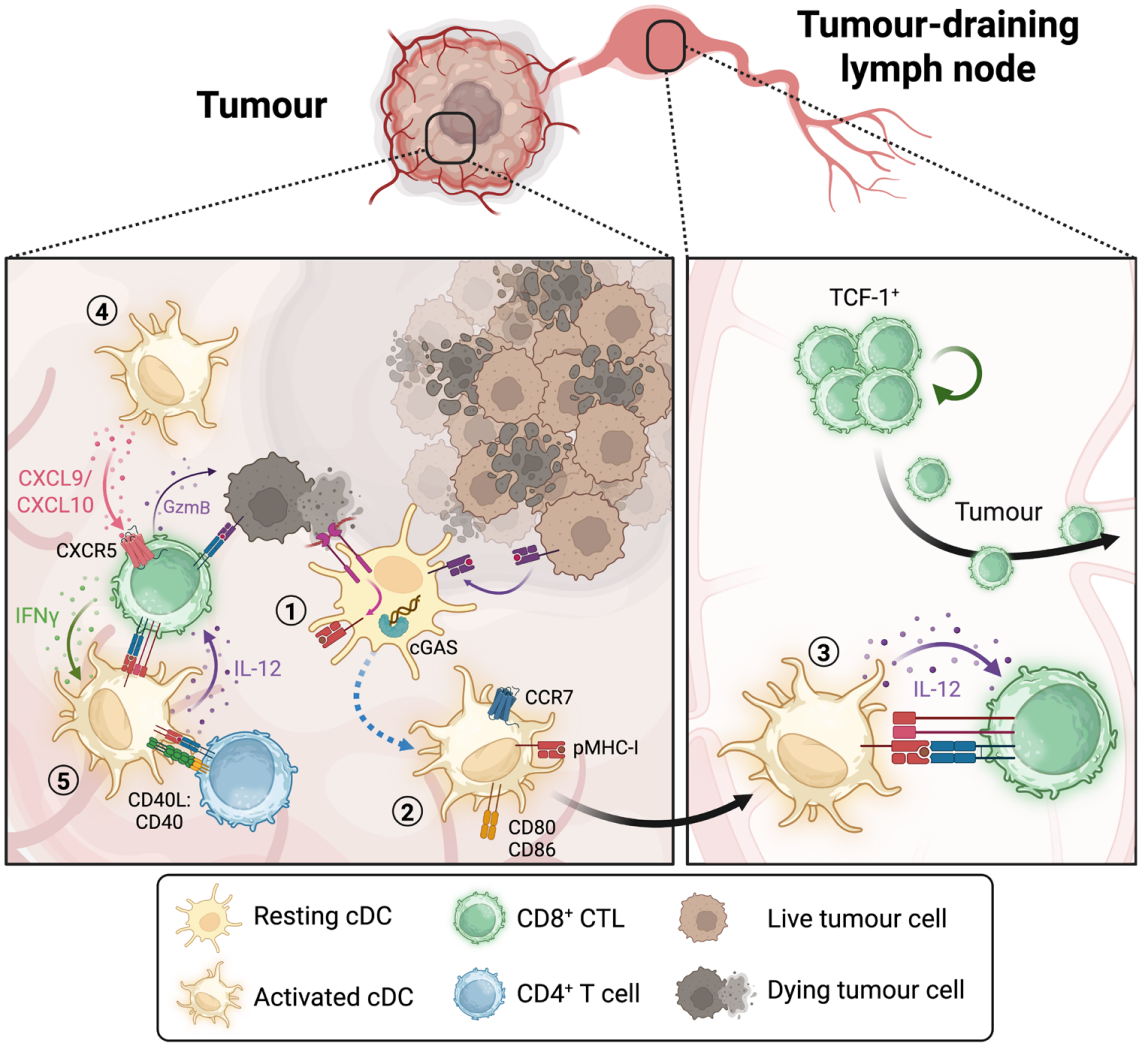
cDCs in anti‐cancer immunity. Resting cDCs sample their environment within the tumour (1) where they acquire tumour antigens from cell corpses through the engagement of phagocytic receptors, which are either common or specific to the cDC subset (e.g., DNGR‐1 in the case of cDC1) or via cross‐dressing of MHC‐I:peptide complexes directly from tumour cells. Engagement of PRRs (e.g., cGAS‐STING) by DAMPs (e.g., tumour DNA) leads to cDC activation and acquisition of an activated phenotype (blue dotted line). Activated cDCs (2) upregulate MHC‐II, co‐stimulatory molecules (CD80 and CD86) and CCR7, which guides their migration to tumour‐draining lymph nodes. In lymph nodes, cDCs interact with CD8^+^ T cells (3), where they present tumour antigens and provide soluble factors such as IL‐12 to prime CD8^+^ T cells. Activated CD8^+^ T cells in lymph nodes maintain a “stem‐like” phenotype involving expression of transcription factor TCF‐1. TCF‐1^+^ CD8^+^ T cells are not fully primed and migrate to the tumour to receive additional co‐stimulatory signals to drive their final effector programme. Tumour‐homing cDCs provide chemoattractants such as CXCL9 and CXCL10 (4) to drive CD8^+^ T cell homing to tumours via CXCR5. (5) CD4^+^ T cells licence via CD40:CD40L interactions to enable IL‐12 production that is necessary to drive full CD8^+^ T cell effector programming. Fully activated CD8^+^ T cells then kill tumour cells in an antigen‐specific manner via production of granzyme B (GzmB). In turn, activated CD8^+^ T cells produce IFNγ that acts back on cDCs to maintain their activation, prolonging the CD8^+^ T cells cytolytic activity against tumour cells for longer periods.

The ability of cDCs to promote cancer immunity is attributed to the priming of anti‐tumour CTL responses (Figure 2). Following the uptake of tumour‐derived material, activated cDCs acquire the expression of the chemokine receptor CCR7, facilitating their migration to tumour‐draining LNs [42, 43] (tdLNs). Upregulation of CCR7 by tumour‐resident cDC1s and cDC2s can be accompanied by shared heterogeneous transcriptional activation programmes that are associated with the activation or suppression of T cell effector function [44, 45]. A fraction of CCR7^+^ cDCs upregulates molecules associated with effector functions, including antigen presentation, co‐stimulation and T cell polarising cytokine production [45] (Figure 2). Such activated cDC1s migrate and deliver neoantigens from the tumour parenchyma to tdLNs, following CCR7 ligand gradients, facilitating the priming of naïve CD8^+^ T cells [42]. cDC1‐primed CD8^+^ T cells in the tdLNs retain a TCF1^+^ stem‐like state and subsequently complete their effector programming by emigrating to the tumour, where they receive additional co‐stimulatory signals including IL‐12 [46] (Figure 2). The homing of activated tumour antigen‐specific CD8^+^ T cells to the tumour microenvironment (TME) is mediated by chemokines CXCL9 and CXCL10 that are produced, among other cells, by tumour‐resident cDC1 [47]. Intratumoural stem‐like CTLs located close to the tumour vasculature transition to a CXCR6^hi^ effector‐like state by interacting with CCR7^+^ cDCs expressing CXCL16, as well as the cytokine IL‐15 that facilitates T cell survival and clonal expansion [48]. In contrast, CCR7^−^ cDC1s further contribute to the acquisition of the CD8^+^ T cell effector programme and support their tumoricidal activity through the secretion of IL‐12, contributing to productive anti‐cancer immunity [48]. In addition, some CCR7^+^ cDC1s remain in the tumour and express immunoregulatory molecules including programmed death ligand 1 (PD‐L1), suppressing anti‐tumour CD8^+^ T cell activity via PD‐1 [45]. This PD‐L1/PD‐1 axis of CTL suppression can be reverted following ICB therapy with anti‐PD‐1. Despite its immunosuppressive role on T cells, PD‐L1 on DCs is essential for their trafficking to tdLNs, via its interaction with CD80 in cis, to facilitate intracellular signalling that controls cell motility [49]. Therefore, the design of new PD‐L1 antibodies for cancer treatment should be considered to exclusively inhibit PD‐L1 trans interactions while sparing the cis interaction with the CD80‐epitope, allowing for both T cell priming and prolonged activation.

Various studies using mice lacking cDC1 provide plenty of evidence for the indispensable role of these cells in promoting anti‐tumour CTL responses [19, 43, 47, 50, 51]. However, there are some reports of functional redundancy of cDC1 for the generation of anti‐cancer CD8^+^ T cell responses in certain contexts. For instance, IFN‐I, either produced locally by tumour cells or when administered systemically in mice, can induce a cDC2 activation state that is characterised by the expression of interferon‐stimulated genes (ISG^+^ cDC2), conferring their capacity to stimulate tumour‐reactive CTL responses in a dominant fashion [24]. Furthermore, during poly(I:C) and anthracycline therapy, anti‐tumour CTL responses are independent of cDC1 and are mediated by other myeloid phagocytes [24, 52, 53].

Beyond CD8^+^ T cell priming, cDCs also impact tumour control by communicating with other immune cell types of adaptive and innate immunity. Secretion of CXCL9, CXCL10 and IL‐12 by cDC1 may promote the accumulation and tune the function of natural killer (NK) cells and group 1 innate lymphoid cells (ILC1s) in the TME [54]. Furthermore, cDC1‐mediated production of IL‐12 permits the control of lung metastasis by NK cells [55]. Both cDC1 and cDC2 exhibit conserved functions in priming CD4^+^ T cells that in turn contribute to the activation of anti‐cancer CTL responses via the secretion of proinflammatory mediators [36, 39, 41]. Interestingly, in some instances, CD4^+^ T cells have been shown to exhibit cytolytic activity similar to that of CTLs. However, it remains unclear whether the acquisition of this cytotoxic effector function results from interactions between naïve CD4^+^ T cells and a specific cDC subset [56].

## 4 Dendritic Cell Function in Cancer Immunity: *Nature* vs *Nurture*

### 4.1 Nature: Cell Intrinsic Determinants of DC Function

In their resting state, cDCs exhibit an elevated phagocytic capacity and high expression of innate immune receptors, allowing them to sample their environment and sense molecular cues leading to cDC activation [9]. Of note, the terms cDC “activation” and “maturation” have been used interchangeably in the literature. Historically, cDC maturation referred to the acquisition of immunogenic T cell priming by cDCs following engagement of pattern recognition receptors (PRRs) by microbial stimuli and downstream signalling [57]. Signalling downstream of PRRs induces the expression of CCR7, co‐stimulatory molecules (including CD80, CD86 and CD40) and production of cytokines by cDCs, which are necessary for migration to LN and naïve T cell priming [58]. However, activated cDCs can also promote antigen unresponsiveness by suppressing immunogenic T cell responses [59, 60, 61]. Here, the term “cDC activation” will be used to describe more broadly the gene expression programmes in cDCs that dictate their ability to promote either immunogenic/effector or tolerogenic/regulatory antigen‐specific T cell responses. Presentation of tumour‐derived antigen to CD8^+^ T cells by cDCs is termed cross‐presentation, a process that involves acquisition and processing of exogenous antigens that are presented in a complex with MHC‐I molecules to naïve CD8^+^ T cells [9, 62]. Binding of the MHC‐I:peptide complex to the T cell receptor (TCR) (signal 1) is not sufficient to stimulate T cell proliferation and effector functions. To do this, activated cDCs must additionally provide co‐stimulation via CD80/CD86 binding to CD28 and CD40 to CD40L (signal 2) and soluble factors (e.g., IL‐12) that drive T cell polarisation towards a particular effector fate (signal 3) [9, 62].

The high prevalence of cell corpses in the TME arises from the induction of cancer cell death following cellular stress and/or treatment with anti‐cancer therapies. Dying cells provide a fruitful source of tumour cell antigens and damage‐associated molecular patterns (DAMPs), some of which are also innate immune receptor agonists that can elicit cDC activation. Innate immune receptor triggering by DAMPs can trigger cDC secretion of cytokines that further amplify cDC activation in a paracrine manner and enhance their ability to prime T cell responses. For example, tumour DNA fragments, exposed from dying tumour cells, are sensed by the cytosolic sensor cyclic GMP‐AMP synthase (cGAS) to activate stimulator of interferon genes (STING)‐dependent IFN‐I production in CD11c^+^ cells, potentially including cDC, to promote robust anti‐tumour T cell responses [63]. Induction of the intratumoural cDC1 activation module that dictates anti‐tumour function is governed by enhanced activity of the transcription factors NF‐κB and IFN regulatory factor 1 [64] (IRF1). Inactivation of the NF‐κB‐IRF1 axis in cDC1 dampens the expression of IFNγ‐responsive genes following activation, compromising their ability to trigger tumour‐reactive CTL responses [64].

The ingestion of dead cell‐associated tumour material is dependent on receptor‐mediated cellular uptake mechanisms that are present in all phagocytes. Despite their scarcity in the TME, migratory CD103^+^ CCR7^+^ cDC1s are the dominant phagocytic population that carries intact antigen to the tdLNs to stimulate antigen‐specific CTL priming [42]. This suggests that cDC1s may harbour specialised mechanisms that allow them to capture and effectively process antigenic material from cell corpses. Indeed, cDC1 are vastly superior at the uptake of cell‐associated antigen as compared to cDC2, which facilitates their acquisition of tumour‐derived antigen for cross‐presentation [65, 66, 67, 68]. DNGR‐1 is a type II transmembrane C‐type lectin receptor that is exclusively expressed on cDC1. DNGR‐1 binds to F‐actin exposed on necrotic dead cell corpses and diverts phagocytic cargo to the cross‐presentation pathway allowing for loading of tumour cell antigen onto MHC‐I and presentation to CD8^+^ T cells [69, 70]. DNGR‐1 plays a redundant role in dead cell uptake and does not affect cDC1 activation, but is indispensable for processing and presentation of dead cell‐associated antigens on MHC‐I molecules post corpse internalisation [71]. DNGR‐1 signalling leads to production of reactive‐oxygen species which disrupt the phagosomal membrane, thereby releasing antigenic cargo to the cytosol, where it can be loaded onto MHC‐I [70]. Similarly, other studies have implicated the membrane pore‐forming proteins perforin‐2 and apolipoprotein L 7C (APOL7C) in the transfer of phagocytic cargo to the cytoplasm, promoting cross‐presentation of cell‐associated antigen by cDC1 [72, 73]. However, the functional relevance of these pore‐forming proteins in the context of immune responses to cancer remains to be confirmed. Endosomal proteins, including WDFY4 and Sec22b, have also been linked to their ability of cDCs to cross‐present tumour antigens to CD8^+^ T cells, although their precise mechanism of action remains elusive [74, 75]. Notably, although WDFY4‐dependent cross‐presentation of cell‐associated antigens for CD8^+^ T cell priming is uniquely attributed to cDC1, WDFY4 can also promote T cell priming of antibody‐coated antigen by both cDC subsets [41]. Furthermore, mouse tumours engineered to express model antigen ovalbumin (OVA) bound to the actin‐cytoskeleton are preferentially controlled in a DNGR‐1‐dependent manner in comparison to those expressing cytoplasmic OVA [76]. In agreement with this, tumour neoantigens associated with the cytoskeleton in mouse and human cancers have been correlated with robust CTL responses and tumour regression [77, 78]. The enhanced control of tumours bearing antigens complexed to F‐actin could be a reflection of targeted compartmentalisation of these antigens to DNGR‐1 positive phagosomes that will in turn result in robust CTL priming. Therefore, biases in receptor‐mediated antigen uptake and phagosomal signalling, which are partly dictated by cDC subset specification, determine the subcellular localisation of tumour antigens and differentially impact antigen processing and presentation and, subsequently, the magnitude of effector T cell responses.

Additional mechanisms of antigen uptake have been recently reported to contribute to the enhanced anti‐tumour properties of cDCs. As compared to tumour‐derived migratory cDC1s, LN resident cDC1s (res‐cDC1), derived from pre‐cDC that directly seed LNs, are not as proficient in promoting CTL priming [42]. This could be attributed to limited access to tumour material by res‐cDC1 and/or differential cDC programming imparted by distinct environmental cues. In support of the latter, recent studies revealed that migratory cDC1 transfer tumour antigens and PRR‐agonists to CD8α^+^ res‐cDC1 through direct physical interactions in the tdLN, enabling them to participate in anti‐tumour CD8^+^ T cell priming [79, 80]. Interestingly, the quality of the CD8^+^ T cell activation profile was different following presentation of tumour antigens by resident or migratory cDC1s [80]. It therefore remains to be clarified whether cDCs generate qualitatively distinct anti‐tumour T cell responses depending on their original location (tumour vs. LN). Furthermore, in certain transplantable tumour models, cDC1s and IFN‐I‐activated cDC2s can directly acquire and present intact tumour‐derived MHC‐I:peptide complexes to CD8^+^ T cells in a poorly defined process called “MHC‐I cross‐dressing” [24, 40]. Therefore, cross‐dressing is a conserved mechanism by which cDC subsets induce CTL priming and establish anti‐cancer immunity whilst bypassing the processing of dead cell‐associated antigens [24, 40].

### 4.2 Nurture: Cell Extrinsic Determinants of DC Function

**FIGURE 3:**
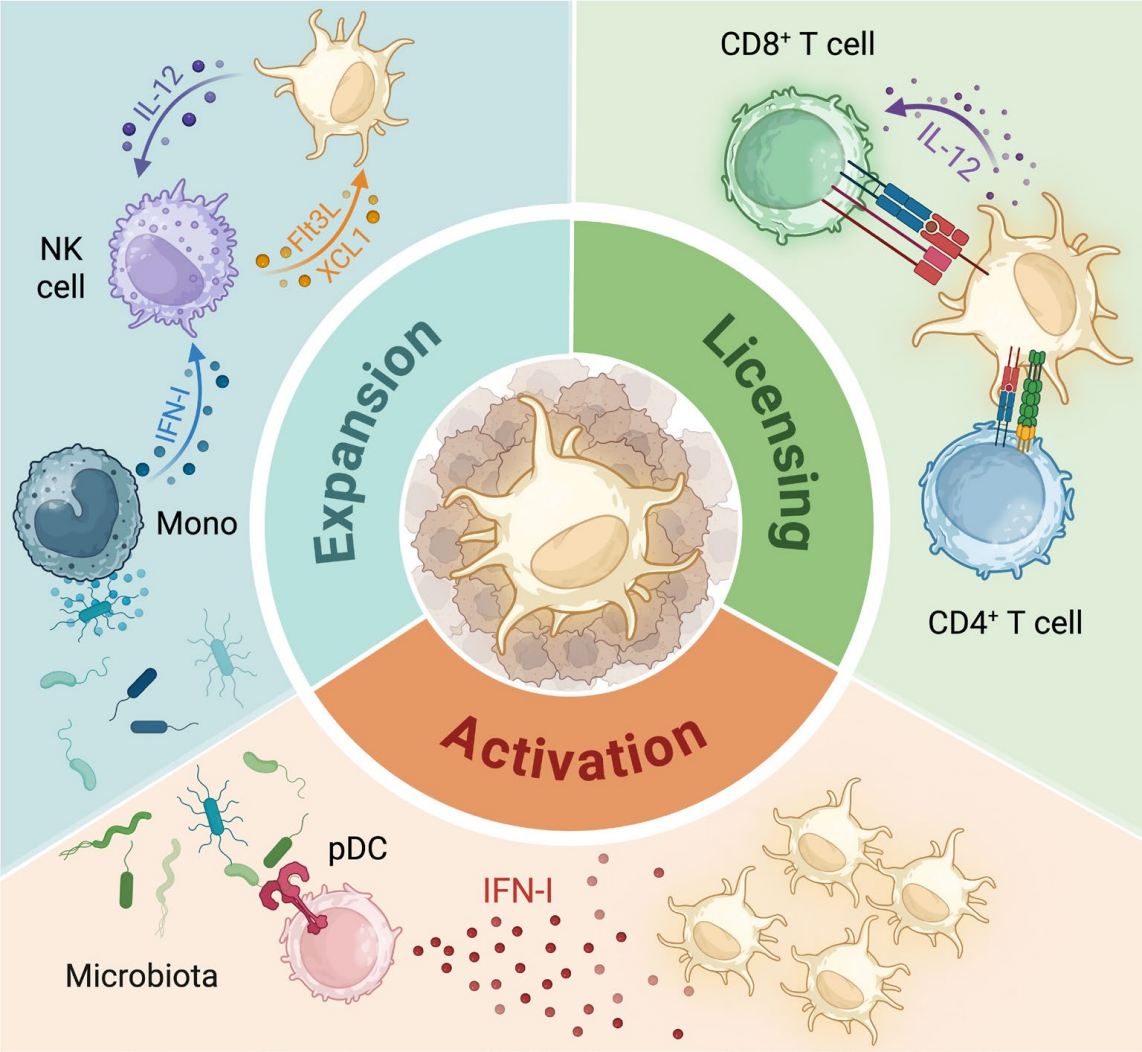
Cell extrinsic regulators of cDC function in cancer. NK cells produce XCL1 and Flt3L to recruit and induce proliferation of cDCs or cDC progenitors, respectively, in the tumour bed. The accumulation of NK cells and subsequent interactions with cDCs in tumours are regulated by microbiome‐dependent IFN‐I production (Expansion; blue). Microbiota also stimulates tonic IFN‐I production by pDCs that act on cDCs to sustain their activation and poise their T cell priming capacities at steady state (Activation; yellow). Finally, CD4^+^ T cells licence cDCs, which in turn endow the cDC with the capacity to produce IL‐12 to fully induce the cytotoxic effector programme of CD8^+^ T cells (Licencing; green).

As described above, cDCs are endowed with several cell‐intrinsic features that determine their ability to promote anti‐tumour immunity. However, these features can be strongly influenced by the local presence of other immune populations within the TME, which will be discussed in this section (Figure 3). In mouse melanoma tumours, NK cells promote cDC1 anti‐tumour functions by enhancing their accumulation in the TME via production of cDC1 chemoattractants CCL5 and XCL1 and cDC growth factor Flt3L [15, 34]. Reciprocally, intratumoral activated cDC1 maintain NK cell activity via IL‐12 production, therefore prolonging cDC1‐NK cell interactions and downstream anti‐cancer immune responses [55]. Expression of NK cell‐associated signatures, *CCL5* and *FLT3L*, positively correlate with cDC1 signatures and overall survival of patients in a variety of cancer subtypes [15, 34].

As mentioned previously, cDC secretion of T cell polarising cytokines is essential for priming CTL responses. For this, CD4^+^ T cells play a critical role in licencing cDCs to produce critical soluble factors including IL‐12 and IFN‐I and surface molecule CD70 that targets and contributes to CTL responses [81]. The process of cDC licencing historically refers to the engagement of the co‐stimulatory molecule CD40 expressed on cDCs with its cognate ligand CD40L that is present on CD4^+^ T helper 1 (Th1) cells. However, it is also possible that additional molecular interactions may occur between these cells [81]. Surface expression of CD40L on CD4^+^ T cells requires the presentation of MHC‐II restricted antigens by cDCs [39, 81]. Following CD4^+^ T cell priming, they can in turn licence cDCs, which enables anti‐tumour CD8^+^ T cell responses [39]. A recent study demonstrated the formation of CD4^+^ and CD8^+^ T cell interactions with the same cDC in tumours, forming a T cell‐cDC triad [82]. Formation of this T cell‐cDC triad cluster is required to enable CD8^+^ T cell cytotoxic function against cancer cells and determines the success of T cell‐based therapies [82]. In human cancers, cDC1 gene signatures associated with CD4^+^ T cell help were positively correlated with overall survival and response to immunotherapy [83]. Additionally, IFNγ production by CD8^+^ T cells in response to anti‐PD‐1 acts on cDC1s in a positive feedback loop, to maintain their activation, allowing cDC1 to support CD8^+^ T cell effector function for longer periods [84].

cDCs can additionally be influenced by host physiology and environmental factors (Figure 3). For instance, the impact of the host microbiome in the modulation of cDC function is a rapidly emerging field. At steady state, microbiome‐dependent PRR triggering on pDCs leads to tonic production of IFN‐I that shapes the epigenetic and metabolic state of cDCs, which is necessary to maintain their T cell priming capacity against peripheral antigens [85]. In the TME, activation of STING in monocytes by microbiota‐derived ligands boosts intra‐tumoural cDC numbers in an IFN‐I‐dependent manner and contributes to NK cell activation [86]. Finally, a recent study also suggested that circadian rhythms can regulate the expression of the co‐stimulatory molecule CD80 on cDC1, thereby tuning their ability to prime anti‐tumour T cell responses at particular times of day [87].

## 5 Natural Barriers to Dendritic Cell Function Hijack Cancer Immunity

**FIGURE 4:**
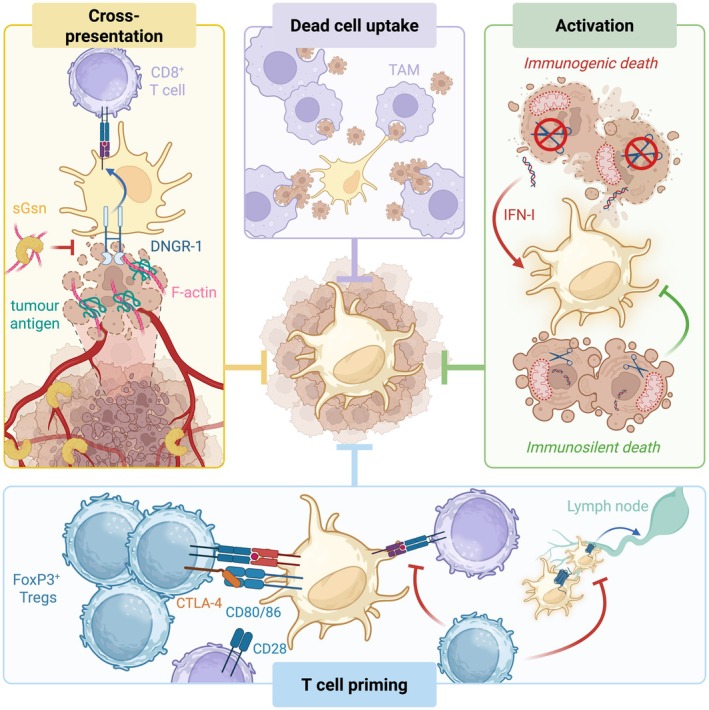
Natural barriers to cDC function in cancer. Several endogenous host factors that contribute to the maintenance of tolerance can also act as natural barriers to cDC function in cancer. cDC1 can recognise F‐actin exposed by dead tumour cell debris via binding to DNGR‐1, which can couple to the cross‐presentation of dead cell‐associated antigen to CD8^+^ T cells. Secreted gelsolin (sGSN) is a plasma protein that sequesters F‐actin and thereby restricts DNGR‐1‐mediated cross‐presentation of tumour antigen by cDC1 (Cross‐presentation; yellow). Tumour‐associated macrophages (TAMs) are proficient at engulfing dying tumour cells and vastly outnumber tumour cDCs, restricting their access to dead cell‐associated material (Dead cell uptake; purple). The activation of cDCs is regulated by the type of cell death occurring. Caspase activation downstream of mitochondrial outer membrane permeabilization (MOMP) induces apoptosis, which is immunologically silent. In contrast, in the absence of caspases, MOMP triggers a slow lytic form of death accompanied by the production of type I interferons (IFN‐I). IFN‐I and immunostimulatory cellular content induce cDC activation, enabling them to prime T cells (Activation; green). Finally, cDC migration to lymph nodes and CD8^+^ T cell priming is blunted via antigen‐specific interactions with FoxP3‐expressing regulatory T cells (Treg) in tumours. This occurs partially due to CTLA‐4 sequestering of cDC CD80/86, which binds CD28 on CD8^+^ T cells, thereby preventing adequate co‐stimulation of CD8^+^ T cells by cDCs (T cell priming; blue).

Strict regulation of the immune response is necessary to ensure maintenance of self‐tolerance whilst promoting pathogen clearance. As discussed in previous sections, cDCs express receptors that enable them to couple dead cell sensing to CD8^+^ T cell priming [9]. However, these pathways of damage recognition are not specific to cancer cell death and therefore are conditioned by firm negative regulation, presumably as a means of limiting presentation of self‐antigens from dying host cells that would be conducive to autoimmunity. This section will explore some examples of host factors that contribute to the maintenance of tolerance and also act as natural barriers that hijack cDC functions in cancer (Figure 4).

Apoptosis is the most well characterised programmed type of cell death and is frequently triggered in the TME due to activation of tumour suppressor mechanisms by cell stress and/or anti‐cancer therapies [88, 89, 90]. In solid tumours, macrophages vastly outnumber cDCs and rapidly clear apoptotic tumour cells before the latter lose their membrane integrity, therefore restricting cDC access to neoantigens and DAMPs [50]. Macrophages express various receptors, including the MER proto‐oncogene tyrosine kinase (MerTK), that facilitate phagocytosis of phosphatidylserine (PtSer) exposing apoptotic cells [91]. MerTK blockade inhibits apoptotic tumour cell clearance by macrophages, permitting cDC1s to induce an IFN‐I response and subsequent T cell‐dependent tumour control [92].

Nevertheless, even at steady state, recognition of apoptotic cells by cDCs doesn't always lead to T cell priming. During homeostasis, apoptotic cell engulfment induces a cDC immunoregulatory programme that suppresses priming of effector T cell responses and induces the development of immunosuppressive Tregs, thereby promoting tolerance to self‐antigens [93]. This type of homeostatic cDC activation is primarily driven by enhanced efflux of cellular cholesterol, due to increased biosynthesis or cholesterol uptake from dead cell corpses, and is dependent on the phagocytic receptor tyrosine kinase AXL and liver X receptor (LXR) signalling [93, 94]. In a murine orthotopic model of lung cancer, uptake of tumour cells led to reprogramming of cDCs to a cellular state resembling homeostatic activation that have been termed “mature DCs enriched in immunoregulatory molecules” (mregDCs) [44]. This reprogramming was accompanied by a gene signature including both immunoregulatory and immunostimulatory genes, which were identified in murine and human lung cancer lesions [44]. The expression of immunoregulatory genes (e.g., *Cd274, Pdcd1lg2, Cd200*) as well as genes associated with Th2 responses and IL‐4 signalling limited the ability of cDCs to prime CTL and Th1 responses, resulting in increased tumour burden in mice [44]. The role of homeostatically activated cDCs in priming the anti‐tumour T cell response remains unclear and is an active topic of research. It is possible that such cDCs promote generation of Treg and Th2 at the expense of Th1 responses that are necessary for the generation of robust CTL effector functions, thereby limiting anti‐tumour immunity. In tdLNs, Th2‐DC clusters were shown to be associated with poor CTL infiltration and reduced patient survival [95]. It remains to be experimentally confirmed whether and how cDC might promote type 2 immune responses resulting in cancer immune escape.

The notion that apoptosis is an “immunologically silent” mode of cell death that limits immunogenic cDC activation [62, 96] has been recently revisited. A series of studies showed that the release of mitochondrial DNA and proteins during mitochondrial outer membrane permeabilization (MOMP), an event that often triggers apoptosis, can lead to the induction of inflammatory mediators such as IFN‐I and CXCL10/9, in a cGAS/STING and NF‐κB‐dependent manner [97, 98, 99]. The inherently proinflammatory nature of dying cells undergoing MOMP is largely silenced by the enzymatic executioners of apoptosis, caspases (cysteine‐aspartic proteases) [97, 98, 99]. However, in pre‐clinical mouse models, the induction of MOMP in a fraction of caspase‐deficient tumours following radiotherapy resulted in pronounced tumour rejection dependent on cDC1‐CD8^+^ T cell action [100]. Similarly, the triggering of necroptosis, a programmed form of lytic cell death that is silenced by caspase‐8, was shown to elicit robust cDC1‐mediated CTL priming and responses against transplantable tumours, which were NF‐κB‐dependent and required cytokine production by dying cells [96, 101]. In addition to intracellular regulation, the immunogenicity of dying tumour cells is also controlled by soluble factors that constrain cDCs from priming T cells. Secreted gelsolin (sGSN), a highly abundant plasma protein, was recently identified as an extracellular checkpoint of cross‐presentation of dead cell‐associated antigens by cDC1 [76]. By sequestering F‐actin from dead tumour cells, sGSN dampens DNGR‐1‐dependent cross‐presentation and limits cDC1‐T cell‐mediated host resistance to cancer. Genetic deletion of *sGsn* increased anti‐tumour T cell responses and boosted the efficacy of immune‐checkpoint blockade and other anti‐cancer therapies in a DNGR‐1‐dependent manner [76]. Additionally, the E3 ligases Cbl/Cblb limit the ability of cDC1 to cross‐prime T cells by suppressing DNGR‐1 signalling [102]. However, paradoxically, a recent study showed that DNGR‐1 limits immune responses to transplantable tumours overexpressing Flt3L, suggesting a context‐dependent role for DNGR‐1 in anti‐cancer immunity beyond cross‐presentation [103]. Collectively, both the adjuvanticity and the antigenicity of dying cells are strictly controlled by a variety of evolutionarily conserved molecules of vertebrate's physiology. This might reflect the adaptation of the host to dampen cDC‐dependent stimulation of harmful autoreactive T cells coming at the expense of robust anti‐tumour T cell responses.

The immunostimulatory function of cDCs can also be suppressed in a cell extrinsic manner as a result of their communication with other immune subsets. Direct interaction of Tregs with either cDC1s or cDC2s in tumours decreases antigen trafficking to LNs and antagonises interactions with Th1 and CD8^+^ T cells, resulting in poor CTL priming and concomitant cancer immune evasion [36, 104, 105, 106]. Key to the restrained anti‐tumour activity of cDCs by Tregs is their tissue‐specific spatial interactions. cDC‐Treg interactions that inhibit CTL priming were found to occur in lung and colorectal mouse cancers in spatially discrete microniches within the tdLNs or the surrounding perilymphatic areas, respectively [105, 106]. Furthermore, activated CXCR3^+^ Tregs were found in various tumours and shown to co‐localise with CXCL9^+^‐cDC1s in the TME of mouse transplantable tumours [104, 107, 108, 109]. Genetic deletion of CXCR3 in Tregs led to a marked increase of cDC1‐mediated cross‐presentation of tumour antigens to CD8^+^ T cells in the TME [104]. Interestingly, beyond their immunomodulatory capacity through secretion of soluble factors, CD4^+^ T cells were also shown to influence anti‐tumour immune responses by exhibiting effector cytotoxic activity. Recently, an unconventional Treg population expressing granzyme B, perforin and LILRB4, induced by cDC2s and monocytes following high doses of MHC‐II:neoantigen vaccines, was found to kill cDC1s, driving cancer immune escape and resistance to ICB therapy [56].

Lastly, several studies underline the impact of microenvironmental changes within or outside the TME as regulators of cDC capacity to mount adaptive immune responses. Hypoxia is a common feature of advanced tumours due to regional insufficiency of vascular oxygen supply of the TME. Prolonged limited oxygen exposure was shown to pose a dysfunctional immunosuppressive metabolic state in intra‐tumoural dendritic cells, limiting their antigen sampling capacity and migration to tdLNs [110, 111]. Moreover, age‐related impairment of host physiology has been shown to severely impact immune function. A recent study demonstrated that aged cDC exhibit impaired activation, weak migratory properties and poor T cell priming capacity, all of which were associated with reduced cancer resistance and weakened response to immunotherapy [112]. Further research is required to understand how age‐associated host functions, as well as related environmental factors, including changes in hormone production, microbiome and nutrition, regulate cDC function in cancer.

## 6 Impairment of Dendritic Cell Function by Cancer Cells

Tumour intrinsic and/or tumour‐derived factors can promote immune evasion in a multifactorial manner by impairing the accumulation and activation of cDCs as well as their ability to sample antigens and stimulate T cells [113]. For instance, tumours with active β‐catenin signalling have reduced CCL4 production, leading to decreased intratumoural cDC1 infiltration and increased tumour growth [114]. Moreover, a cancer‐derived prostanoid prostaglandin E_2_ (PGE_2_) is a major driver of pro‐tumorigenic inflammation, driven in part by reduced cDC1 infiltration in the TME [15, 115, 116]. PGE_2_ acts on EP2 and EP4 receptors on NK cells to supress cancer‐inhibitory inflammation and tumour reactive CTL responses, favouring tumour progression and resistance to immunotherapy in mice [116]. In human cancers, a PGE_*2*_‐related proinflammatory gene signature inversely correlates with patient overall survival response to ICB therapy, highlighting the relevance of PGE_2_ synthesis and downstream targets as an immunomodulatory hub that is predictive of clinical outcome [116].

Malignant transformation itself often suppresses the phagocytosis of dead cells and subsequent presentation by cDCs, diminishing effector T cell responses. As an example, PtSer‐binding receptor TIM4 is downregulated on lung resident cDC1 during cancer development, impairing CTL‐dependent control of malignant cells [117]. Moreover, SIRPα acts as a cell intrinsic barrier to cDC2 activation following its binding to CD47, a key “do not eat me” signal expressed by cancer cells. SIRPα‐CD47 ligation promotes phagocytic degradation of ingested mitochondrial DNA present in corpses [118]. Inhibition of this interaction permits cGAS activation and subsequent IFN‐I production that enabled cDC2 to support anti‐tumour immune responses [118]. Furthermore, tumour‐derived sGSN is sufficient to drive cancer immune evasion by limiting T cell cross‐priming by cDC1. In alignment to this, intratumoural expression of *sGSN* inversely correlates with overall survival in several human cancers [76].

Finally, cancer cells can negatively regulate antigen presentation by alternating the lipid metabolism of antigen‐presenting cells (APCs). Lipid peroxidation by‐products induce endoplasmic reticulum stress in cDCs, which result in the formation of lipid bodies hindering the trafficking of MHC‐I:peptide complexes to the cell surface and therefore diminishing T cell activation [119, 120]. Metabolic bioproducts of cancer metabolism can direct a dysfunctional state in cDCs, disarming their ability to orchestrate cytotoxic anti‐tumour T cell responses. Cholesterol efflux in response to LXR ligands derived from tumour cells has been shown to promote cancer immune escape by inhibiting DC migration to the tdLN through downregulation of CCR7 [121]. PGE_2_ can also act directly on cDC1s, leading to downregulation of the transcription factor IRF8 and downstream transcriptional modules that are necessary for establishing T cell immunity to cancer [122]. Collectively, depending on the oncogenic drivers and evolutionary trajectory of tumours as well as their interaction with the surrounding stromal cells, cancer cells adapt to hijack cDC function and thereby evade immune control.

## 7 Targeting Dendritic Cells to Boost Cancer Immunity

**FIGURE 5:**
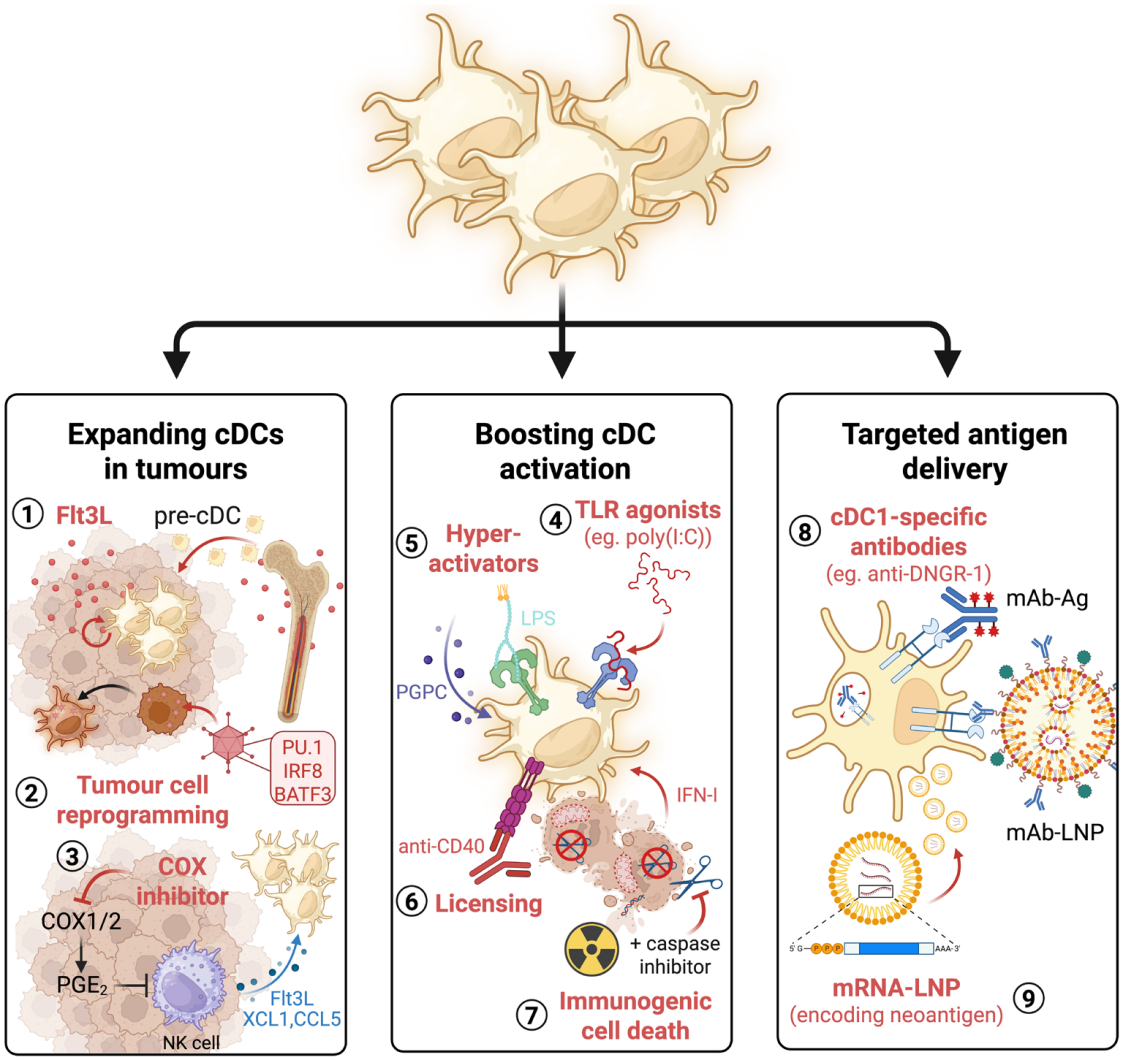
Strategies to target cDC function to boost anti‐cancer immunity. The scarcity of cDCs in tumours can be overcome by various strategies aiming to boost cDC numbers in tumours, such as exogenous administration of Flt3L (1) which elicits cDC proliferation and pre‐cDC recruitment to tumours, reprogramming of cancer cells into cDCs by delivering cDC transcription factors with an adenoviral vector (2) or inhibition of cyclooxygenases (3), which produce prostaglandin‐E2, a negative regulator of the NK‐cDC1‐T cell axis. Enhancing cDC activation in tumors can also be achieved by administration of TLR agonists (4), including poly(I:C) which binds TLR3 on cDC1, or provision of lipopolysaccharide (LPS) and oxidised lipids such as PGPC that together induce cDC hyperactivation (5), characterised by enhanced production of IL‐1β. Alternatively, anti‐CD40 (6) can be used to licence cDCs, bypassing the need for licencing by CD4^+^ T cells. Induction of immunogenic cell death (7) can be achieved by irradiation combined with caspase inhibition, which elicits production of type I interferon (IFN‐I) by dying cells that activates cDCs. Finally, antigen (Ag) can be delivered to intra‐tumoural cDCs by conjugating antigen to cDC1‐specific monoclonal antibodies (mAb) (8), such as anti‐DNGR‐1. In the same way, mAb‐conjugated lipid nanoparticles (LNP) can be delivered to cDCs, which can be made to carry antigen alone or antigen plus adjuvant, such as STING agonists, to additionally boost cDC activation. Finally, LNPs carrying mRNA encoding tumour neoantigens (9) permit translation of neoantigen within the cDC, facilitating loading of neoantigen‐derived peptides onto MHC‐I for presentation to CD8^+^ T cells.

cDCs represent a key determinant of T cell function in cancer. Therefore, several strategies have been proposed to harness cDC biology as a means of overcoming inherited or acquired resistance to T cell‐based immunotherapies (Figure 5). Comprehensive summary tables of all clinical trials to date using DC‐based immunotherapies and their outcomes have been previously described in detail [113, 123]. Below, I describe the different strategies used so far to boost DC activity and highlight their therapeutic efficacy in pre‐clinical and clinical studies.

Adoptive transfer of autologous neoantigen‐loaded cDCs has been used as a strategy to boost presentation of tumour‐specific antigens and enhance T cell immunity to cancer [113]. However, there is a major bottleneck in generating cDC vaccines due to the scarcity of cDCs and their progenitors in human peripheral blood [124]. In contrast, Mo‐DC vaccines can be easily manufactured from highly abundant circulating monocytic progenitors and were used in numerous clinical trials as an alternative approach to deliver tumour antigens and prime cancer‐specific T cell responses [113]. However, Mo‐DC‐based vaccines exhibit relatively low clinical benefit that may be largely attributed to their relatively poor T cell priming potency relative to cDCs [23].

A plethora of studies in pre‐clinical models has highlighted promising strategies that significantly enhance the efficacy of T cell‐based immunotherapies by modulating the abundance and activity of cDCs in situ [113, 123] (Figure 5). Systemic administration of Flt3L, as a single agent or in combination with cDC activators (poly(I:C) or anti‐CD40), successfully increases cDC1 numbers in the TME and augments responses to ICB therapy in mouse cancers [43, 125]. Delivering tumour‐homing viral vectors or bacteria encoding *Flt3l* resulted in prolonged release of FLT3L in the TME accompanied by strong anti‐tumour immunity in pre‐clinical models [51, 126]. Similar therapeutic outcomes were obtained following transfer of irradiated cancer cells or cancer‐specific T cells that were engineered to express FLT3L [127, 128]. Targeting PGE_2_ biosynthesis or signalling, using cyclooxygenase‐2 inhibitors or EP2/4 antagonists respectively, substantially increased responses to ICB therapy in mouse transplantable tumours by enhancing cDC1 infiltration and activity [115, 129].

Boosting cross‐presentation of tumour antigens by cDC1 can restore T cell immunity to cancer and improve immunotherapy efficacy (Figure 5). Some of the most efficacious strategies in this regard include the use of antibody‐based blockade of macrophage factors that outcompete cDC dead cell uptake (e.g., anti‐MertK) and cDC1 activation (e.g., anti‐TIM3 and anti‐CD47) [92, 118, 130, 131]. Triggering of immunogenic cell death can enhance T cell cross‐priming by increasing cDC1 activation [96, 97, 100, 101]. Furthermore, administration of lipopolysaccharide (LPS) in the presence of oxidised phospholipids was shown to shift cDC1s from an ageing‐related dysfunctional state to a hyperactivation programme characterised by expression of co‐stimulatory molecules, enhanced migration to lymph nodes and prolonged inflammasome‐mediated IL‐1β secretion. Hyperactivated cDC stimulate protective responses accompanied by enhanced cancer resistance through induction of durable effector and memory T cell responses [112, 132, 133]. A recent in vitro screen using an immortalised cDC1 line identified BCL2 as a negative regulator of T cell priming by cDC1 [134]. Pharmacological or genetic targeting of BCL2 enhanced cDC1 activation, boosting their cross‐presentation capacity and sensitising orthotopic mouse tumours to ICB therapy [134].

Furthermore, of particular interest, are recent interventions that specifically target the cDC‐T cell axis, improving anti‐tumour immune responses in pre‐clinical models (Figure 5). Administration of bispecific antibodies that bind to surface molecules expressed on cDCs (e.g., DNGR‐1, CD40, PD‐L1) and T cells (e.g., PD‐1, CD40L, CD28) enhances cDC‐T physical interactions, restoring anti‐tumour immunity in animal models [135, 136, 137, 138, 139]. Similarly, STING agonists incorporated within lipid nanoparticles (LNP) that target DNGR‐1 stimulate cDC1 to promote anti‐tumour immune responses and enhance the response to ICB in mice [140]. Following very promising results in pre‐clinical models, multiple ongoing clinical trials are currently testing the therapeutic efficacy of several STING agonists in human cancers [141, 142]. LNPs have also been used to deliver neoantigens to cDCs, improving T cell‐based immunotherapies in mouse models [143]. Tumour antigen‐conjugated antibodies that target DNGR‐1 promote robust T cell‐mediated tumour control in mice by directing antigen processing to phagosomal signalling that favours T cell cross‐priming [144]. Notably, recent studies highlight that cross‐presentation is not just a process restricted to cDC1s but can be inherited in heterologous cells including tumour cells, through the expression of receptors or transcription factors that drive this process, fortifying them to restore T cell immunity to cancer [70, 145, 146]. However, ex vivo reprogramming and reinfusion of cDC‐like cancer cells present significant challenges for clinical application due to the scarcity of circulating cDCs in humans. This limitation was overcome in transplantable tumours in mice using adenoviral delivery of transcription factors that facilitate the conversion of cancer cells to cDC1‐like cells. As little as 2% of cancer cell reprogramming was sufficient to establish systemic anti‐tumour immune responses and increase the responses to ICB therapy [147]. Therefore, enabling cDC function in cancer cells in situ offers an exciting opportunity for the development of novel immunotherapies for human cancers that go beyond the margins of DC‐based therapies that could disable key immune evasion mechanisms such as cancer heterogeneity, loss of antigen presentation in cancer cells and cDC scarcity.

In summary, various strategies with the aim of controlling cDC anti‐tumour properties in pre‐clinical models have paved the way for the design of new therapeutic strategies. Some of these strategies have been shown to boost cancer immunity either as single agents or in combination with other anti‐cancer therapies. Although the initial trials using DC vaccines showed limited success in clinical practice, newly developed strategies targeting cDCs and their interactors in situ have demonstrated remarkable therapeutic potential in mouse models of cancer. It remains to be assessed whether targeting the activity of cDCs could be beneficial in human cancers as monotherapy or in synergy with existing immunotherapies. Furthermore, it is crucial to evaluate whether DC‐based therapies compromise self‐tolerance, thereby increasing the risk of autoimmunity and chronic inflammatory diseases, as previously observed in T cell‐based immunotherapies [148]. Thus, further characterisation of the proposed cDC‐based therapies is needed to select for those that ensure optimal effector T cell responses with minimal adverse effects.

## 8 Concluding Remarks

Over 50 years of research since the identification of cDCs has shed light on the remarkable properties of these cells, defining them as indispensable gatekeepers of host defence against pathogens and cancer. Strategies to harness cDC functions in cancer, some of which have been described in this review, are therefore of paramount interest in the development of novel treatments to synergise with current T cell‐based immunotherapies. Recently, single‐cell technologies have revealed previously underestimated heterogeneity among cDCs, particularly cDC2 [14, 23, 149]. As cDC2 are much more numerous than cDC1 in tumours, current efforts to understand the biological function of newly identified cDC2 subsets states will be essential to reveal novel targets for immunotherapies aiming to restore cancer immunity.

Sequencing of human and mouse tumours has revealed conserved cDC activation states that extend beyond cDC subset specification [123]. This aligns with numerous studies suggesting that activated cDC subsets have conserved roles in anti‐tumour immunity [36, 39, 40, 41], shifting the current focus from cell subset to cell state. Interestingly, immunogenic (anti‐tumour) and immunoregulatory (pro‐tumour) programmes are often co‐expressed in activated cDCs [44]. Spatiotemporal characterisation of cDC programmes will shed light on how different cDC activation programmes are coupled to each other and reveal potential mechanisms underpinning their transition. Proteomic, metabolomic and epigenetic analyses will further enhance our understanding of cDC states and provide opportunities for their manipulation to boost cancer immunity. While it is clear that in many contexts, cDC1 plays a critical role in sustaining anti‐cancer T cell responses that cannot be compensated by cDC2, the existence of non‐redundant roles for cDC2s in regulating cancer immunity remains to be determined [19, 37, 38]. In this regard, engineering mice to specifically lack cDC2s will complement the existing cDC1‐deficient mouse models and will further help to map non‐redundant functions of cDC subsets and their role in cancer immunity.

The TME is a highly complex ecosystem comprising spatially segregated microniches of cell and molecular interactions that influence the T cell priming functions of cDCs. The interactions of cDCs with NK and CD4^+^ T cells have been shown to be critical for the establishment of anti‐tumour immune responses [15]. cDCs interact with various other immune and non‐immune cell types, including mast cells, basophils, epithelial cells, neurons and fibroblasts, influencing their activity across tissues [9, 15, 16, 17]. However, how these interactions impact anti‐cancer immunity remains unclear. Finally, cDC function is continuously regulated by cancer‐ or host‐specific molecular barriers. Future research aimed at uncovering the mechanisms through which these barriers hijack cDC activity will be crucial for developing therapeutic strategies to manipulate the tumour ecosystem and enhance anti‐tumour immunity.

## Author Contributions

**Evangelos Giampazolias:** conceptualization (lead), writing – original draft (lead), writing – review and editing (lead).

## Conflicts of Interest

The author declares no conflicts of interest.

## Data Availability

The author has nothing to report.
